# Supplementary figures and images for: Calcium current modulation by the γ1 subunit depends on alternative splicing of CaV1.1
Source: J Gen Physiol. 2022 Mar 29;154(9):e202113028. doi: 10.1085/jgp.202113028 (PMC9037348; doi:10.1085/jgp.202113028)

FIGURE 1B

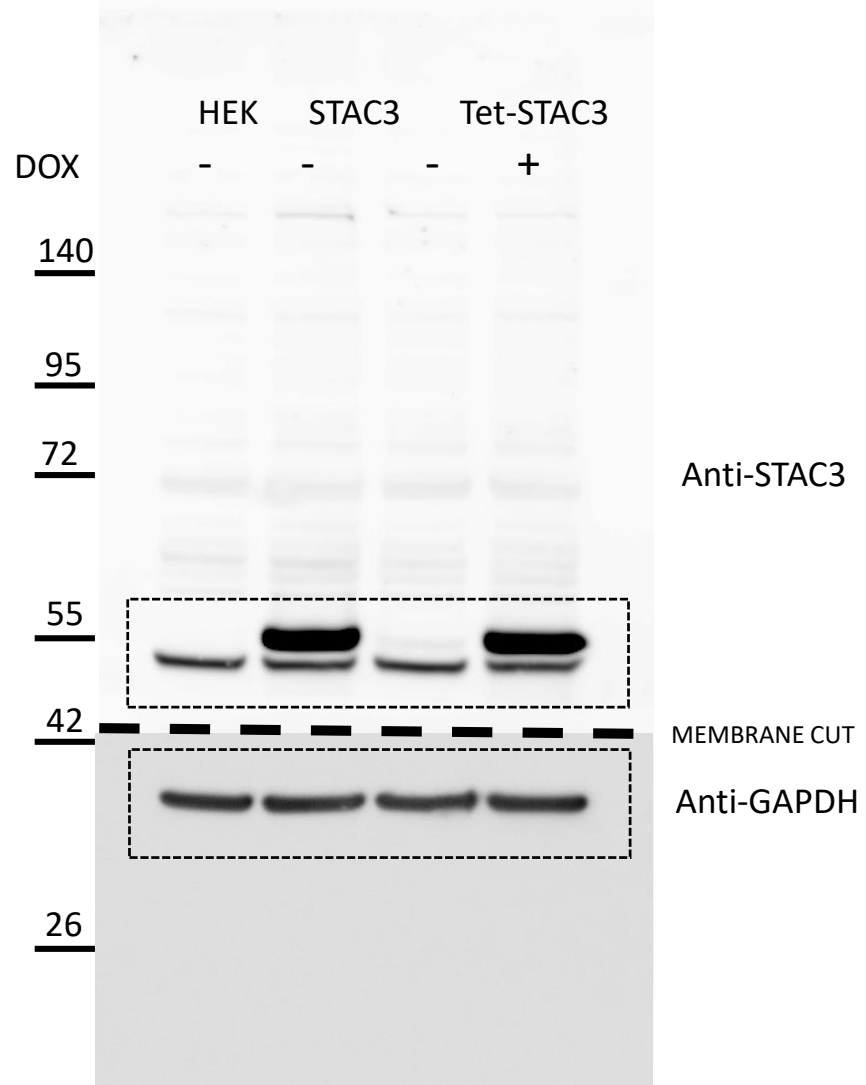

Supplement: SourceData F1 — is the source file for Fig. 1. [file JGP_202113028_SourceDataF1.pdf]
